# Low-affinity neurotrophin receptor p75 of brain-derived neurotrophic factor contributes to cancer-induced bone pain by upregulating mTOR signaling

**DOI:** 10.3892/etm.2020.8528

**Published:** 2020-02-12

**Authors:** Xiao-Wen Meng, Xiao-Hong Jin, Xiang Wei, Li-Na Wang, Jian-Ping Yang, Fu-Hai Ji

Exp Ther Med 18:4379-4387, 2019; DOI: 10.3892/etm.2019.8097

Subsequently to the publication of this article, the authors have realized that the first three authors listed on the paper, Xiao-Wen Meng, Xiao-Hong Jin and Xiang Wei, were not credited as joint first authors who made equal contributions to this study (asterisks should have been included alongside their names to accompany the appropriate footnote to reflect this).

Therefore, the correct author affliations for this article should have been presented as follows:

Xiao-Wen Meng^*^, Xiao-Hong Jin^*^, Xiang Wei^*^, Li-Na Wang, Jian-Ping Yang, Fu-Hai Ji

Department of Anesthesiology, The First Affiliated Hospital of Soochow University, Suzhou, Jiangsu 215006, P.R. China

^*^Contributed equally

The authors regret their oversight in this regard, and apologize for any inconvenience caused.

